# Expert consensus on low-calorie sweeteners: facts, research gaps and suggested actions

**DOI:** 10.1017/S0954422419000283

**Published:** 2020-06

**Authors:** Margaret Ashwell, Sigrid Gibson, France Bellisle, Judith Buttriss, Adam Drewnowski, Marc Fantino, Alison M. Gallagher, Kees de Graaf, Séverine Goscinny, Charlotte A. Hardman, Hugo Laviada-Molina, Rebeca López-García, Berna Magnuson, Duane Mellor, Peter J. Rogers, Ian Rowland, Wendy Russell, John L. Sievenpiper, Carlo la Vecchia

**Affiliations:** 1 Ashwell Associates, Ashwell, Hertfordshire, UK; 2 Sig-Nurture Ltd, Guildford, Surrey, UK; 3 Nutri Psy Consult, Paris, France; 4 British Nutrition Foundation, London, UK; 5 Center for Public Health Nutrition, University of Washington, Seattle, WA, USA; 6 Fantino Consulting SAS, F-69230 Saint Genis Laval, France; 7 Nutrition Innovation Centre for Food and Health (NICHE), Ulster University, Coleraine, UK; 8 Division of Human Nutrition and Health, Wageningen, The Netherlands; 9 Service Organic Contaminants and Additives (SCIENSANO), Brussels, Belgium; 10 Department of Psychological Sciences, University of Liverpool, Liverpool, UK; 11 Escuela de Ciencias de la Salud, Universidad Marista de Mérida, Merida, Mexico; 12 Logre International Food Science Consulting, Mexico City, Mexico; 13 Health Science Consultants, Inc., Mississauga, Ontario, Canada; 14 Aston Medical School, Aston University, Birmingham, UK; 15 Nutrition and Behaviour Unit, School of Psychological Science, University of Bristol, Bristol, UK; 16 Department of Food and Nutritional Sciences, University of Reading, Reading, UK; 17 University of Aberdeen Rowett Institute, Aberdeen, UK; 18 Department of Nutritional Sciences, University of Toronto, Toronto, Canada; 19 Division of Endocrinology and Metabolism, Department of Medicine, St. Michael's Hospital, Toronto, Canada; 20 Li Ka Shing Knowledge Institute, St. Michael’s, Toronto, Canada; 21 Department of Clinical Sciences and Community Health, University of Milan, Milan, Italy

**Keywords:** Low-calorie sweeteners, Weight management, Glucose control, Food safety, Nutrition policy, Consensus Reports

## Abstract

A consensus workshop on low-calorie sweeteners (LCS) was held in November 2018 where seventeen experts (the panel) discussed three themes identified as key to the science and policy of LCS: (1) weight management and glucose control; (2) consumption, safety and perception; (3) nutrition policy. The aims were to identify the reliable facts on LCS, suggest research gaps and propose future actions. The panel agreed that the safety of LCS is demonstrated by a substantial body of evidence reviewed by regulatory experts and current levels of consumption, even for high users, are within agreed safety margins. However, better risk communication is needed. More emphasis is required on the role of LCS in helping individuals reduce their sugar and energy intake, which is a public health priority. Based on reviews of clinical evidence to date, the panel concluded that LCS can be beneficial for weight management when they are used to replace sugar in products consumed in the diet (without energy substitution). The available evidence suggests no grounds for concerns about adverse effects of LCS on sweet preference, appetite or glucose control; indeed, LCS may improve diabetic control and dietary compliance. Regarding effects on the human gut microbiota, data are limited and do not provide adequate evidence that LCS affect gut health at doses relevant to human use. The panel identified research priorities, including collation of the totality of evidence on LCS and body weight control, monitoring and modelling of LCS intakes, impacts on sugar reduction and diet quality and developing effective communication strategies to foster informed choice. There is also a need to reconcile policy discrepancies between organisations and reduce regulatory hurdles that impede low-energy product development and reformulation.

## Introduction and aim of the Consensus Report

A number of reviews, some narrative and some systematic, have discussed the evidence for the safety of low-calorie sweeteners (LCS) and their effects on appetite, food intake, body weight, glucose control and other health outcomes^(1–8)^. Evidence has also been evaluated by authorities, such as the European Food Safety Authority (EFSA), the (US) Dietary Guidelines Advisory Committee, the French Agency for Food, Environmental and Occupational Health & Safety (ANSES) and Public Health England, who have issued statements or opinions on the use of LCS^(9–13)^. Other groups of scientific experts have generated consensus statements, position papers, or other statements on LCS. These include the British Dietetic Association, Diabetes UK, the American Heart Association and American Diabetes Association (AHA/ADA)^(14–20)^.

This paper describes the results of a workshop in which seventeen experts convened to discuss and debate the science and policy relating to the use of LCS. The aims were to establish, via consensus-forming techniques, clear and simple statements on LCS that all the panel agreed (facts), to highlight the areas where more research is required (gaps) and to propose how progress might be achieved (actions). It is hoped that the provision of these statements on safety and potential benefits of LCS will assist health practitioners and policy makers to promote consistent messages and develop strategies based on sound science. Identification of the gaps and actions will help promote better study designs, suggest priorities for research funding and thereby encourage more coherent public health policy.

## Background to low-calorie sweeteners and their regulatory approval process

All LCS have undergone an extensive safety evaluation process by international and national regulatory food safety bodies both before and after their approval for use in the market. The FAO/WHO Joint Expert Committee on Food Additives (JECFA)^(21)^, the US Food and Drug Administration (FDA)^(22)^ and EFSA^(9)^ have confirmed the safety of all approved LCS as food additives. Hence there is an extensive body of evidence from both animal models and human studies that support the safety of LCS. Each compound is considered individually as their characteristics, metabolism and metabolic fates are different^(23)^. Furthermore, there is an ongoing review process to ensure that any new information on safety is evaluated, for example recent scientific opinions by EFSA on aspartame and sucralose^(24,25)^.

As part of the LCS safety evaluations, the regulatory authorities establish the Acceptable Daily Intakes (ADI) for each sweetener^(26)^. The ADI is defined as an estimate of the amount of a food additive, expressed per kg body weight, that can be ingested daily by individuals over a lifetime without appreciable risk to health. ‘Without appreciable risk’ means, based on the current knowledge, ‘certainty that no harm will result, even after a lifetime of exposure to the additive’^(27)^. The current ADI for LCS were established using the ‘no observed adverse effect level’ (NOAEL). This is the highest dietary level of an additive at which no adverse effects were observed in animal studies. The ADI is typically set at 1/100th of the NOAEL (10-fold reduction for inter-species variation and 10-fold reduction for intra-species variation) to give a large margin of safety for even the most sensitive consumer. The ADI refers to a lifelong exposure situation, not a single occasion, and thus infrequent consumption of levels higher than the ADI are not a health concern. Because of the large safety margin used in setting the ADI, it is likely that an ADI for a given additive would have to be exceeded by some considerable amount for an extended period of time for there to be any risk of harm to human health. However, if an intake estimate indicates that the ADI may be regularly exceeded by certain sectors of the population, the regulatory authority may advise a reduction of levels in foods, or to reduce the range of foods in which the additive is permitted for use^(27)^. In some cases, the ADI may be ‘not specified’ when the total potential intake from all possible sources does not represent a hazard to health, and hence no numerical ADI is needed. It should be noted that, in the future, the benchmark dose (BMD) will be the preferred approach for establishing a reference point^(28)^. However, discussion of the expert considerations and data requirements for calculation of a BMD is beyond the scope of this paper.

In relation to efficacy, EFSA has a system for evaluating dossiers of evidence submitted for the substantiation of health claims^(29)^. In 2011, the EFSA Panel on Nutrition, Novel Foods and Food Allergens concluded that there was sufficient scientific evidence to support the claims that intense sweeteners, like all sugar replacers, lead to a lower rise in blood sugar levels after meals if consumed instead of sugars, and maintain tooth mineralisation by decreasing tooth demineralisation; again, if consumed instead of sugars. However, at that time, EFSA’s experts found no clear cause-and-effect relationship to substantiate the claims that intense sweeteners when replacing sugars maintain normal blood sugar levels, or maintain/achieve a normal body weight^(30)^.

There are currently various jurisdiction-specific restrictions on the use of LCS in foods and beverages. For example, under European legislation, sweeteners are only permitted if used to replace sugars for the production of energy-reduced food (i.e. with 30 % less energy), non-cariogenic food, or food with no added sugars^(31)^. This limits the options available to manufacturers for more modest reformulation or stepwise reduction of sugar content in food and drink through the use of sweeteners.

## Methods

The consensus workshop was designed to follow a conference held by the International Sweeteners Association (ISA) in London on 6 November 2018 entitled ‘The science behind low calorie sweeteners: where evidence meets policy’. The panel members were all speakers or chairs at that conference, chosen for their international expertise in LCS science and policy. The workshop was chaired and facilitated by two independent consultants in nutrition science (M. A. and S. G.), who drafted the paper and coordinated responses from participants. The ISA provided funds for the venue and speakers’ expenses. They were observers at the workshop but had no control over the paper. Disclosures of interest for all authors are given.

The workshop leaders (M. A. and S. G.) identified three key themes or topic areas for discussion at the workshop:

(1) Role of low-calorie sweeteners in weight management and glucose control;
(2) Consumption and safety of low-calorie sweeteners and consumer perception;
(3) Role of low-calorie sweeteners in relation to nutrition policy.

As the workshop was time-limited the choice of themes was based on the pertinence in terms of current debates on LCS, and the available expertise represented by the panel.

Before the workshop, each panel member was asked to provide feedback on three questions with respect to their own area of expertise:

(a) Statements of fact: what do we know?
(b) Questions and type of evidence needed (gaps: what do we still need to know?)
(c) How this might translate to further research work or policy (actions: what should we do?).

Comments were minimally edited by M. A. and S. G. to produce the working document with provisional statements/questions/actions for each theme.

At the workshop all the participants discussed the working document in detail. A scoring system (1 = strongly disagree to 10 = strongly agree) was used to evaluate level of agreement on the ‘facts’. Statements that achieved a high level of agreement were discussed further. Participants refined the wording of each statement to reach consensus. Having established agreement on the facts, participants identified the major gaps or research questions. Finally, participants identified the most important ‘actions’ suggested in the working document and these were then summarised. Participants agreed to the process for further review and publication, i.e. that the workshop leaders would circulate the draft consensus document for comments, integrate responses and write the discussion before presenting the final article to all participants for review and approval. Table 1 shows the timeline of the project.

**Table 1.** Timeline of the project

Date	Process
January 2018	Identification of ISA conference speakers and chairs
April 2018	Workshop leaders (M. A. and S. G.) appointed
	Conference speakers and chairs invited to workshop
May 2018	Three key workshop themes identified by workshop leaders
May 2018	Workshop leaders agree questions for experts based on the three themes for workshop
July 2018	Experts asked to provide provisional answers to questions
September 2018	Workshop leaders collate expert comments into working document
6 November 2018	ISA conference
7 November 2018	Expert workshop
November 2018	Draft consensus statements agreed at workshop, circulated to experts
December 2018	Comments received from experts
December 2018–January 2019	Draft paper written by workshop leaders
January 2019	Draft paper circulated to experts for approval
February 2019	Paper finalised and submitted to journal
July and August 2019	Revisions to paper agreed by panel

ISA, International Sweeteners Association.

## Results

The results are given below in the form of the consensus statements for the three themes and the three questions relating to each theme. The panel agreed the most pertinent references to cite for each consensus statement.

### Theme 1: Role of low-calorie sweeteners in weight management and glucose control: the scientific evidence

#### 1a Facts: what we know

(1) When substituted for sugars to reduce energy density of foods and drinks, LCS reduce net energy intake and assist weight management^(3,5,12,13)^.
(2) Intervention studies have shown that beverages containing LCS have at least a similar effect on appetite and energy intake to water^(5,32)^.
(3) The collective evidence supports the conclusion that LCS have no adverse effect on blood glucose and insulin regulation (HbA1c, fasting and postprandial glucose and insulin levels) in individuals with, and without, diabetes^(2,33,34)^.
(4) The potential value of LCS in dietary management of diabetes derives from their role as substitutes for sugars, and hence carbohydrates^(19)^.
(5) Confounding by adiposity, and reverse causality can explain the positive association between LCS and type 2 diabetes and other cardiometabolic diseases, reported in some observational studies^(35–37)^.
(6) Regarding effects involving the human gut microbiota, data are limited and do not provide adequate evidence that LCS influence gut health at doses relevant to human use^(38)^.

#### 1b Gaps: what we don’t know

(1) What are the long-term effects of LCS on glucose tolerance, gut function, cardiometabolic effects, gut microbiota and weight management?
(2) How are these effects altered according to personal factors, such as age, sex, ethnicity, socio-economic status, health status, diet and lifestyle?
(3) How do these effects differ according to dietary context (*ad libitum v.* weight-control diet) and form of LCS (in liquids or solids), and type or blend of LCS?
(4) Does reducing exposure to sweetness have consequences for food choice and intake in the medium to long term?
(5) Can LCS help improve long-term type 2 diabetes management, when part of standard dietary and lifestyle approaches?

#### 1c Actions: what should be done?

(1) There is a need for a portfolio of well-designed randomised controlled trials (with an appropriate time-frame of 1 year or more) with different comparators and different carriers of LCS (food and beverage matrices). The trials should be conducted by level of ‘free sugar’ intake in different populations; they should use multiple endpoints (diet quality, gut microbiota function and metabolomics, and wider health and quality of life measures). They should be done in the context of weight-control diets, including for type 2 diabetes and also in non-restrictive diets.
(2) There is a need for population cohort studies to model changes in weight/cardiometabolic risk in the context of changes in LCS consumption, not baseline LCS values. The studies should include substitution analysis (for example, LCS beverages for energy beverages, water, etc.) and adjustment for adiposity. Their data should be made available for further analysis.
(3) There is a need for a collation of evidence to support future health claim submissions for LCS and body weight control, as data become available.

### Theme 2: Consumption and safety of low-calorie sweeteners and consumer perception

#### 2a Facts: what we know

(1) The safety of LCS is demonstrated by a substantial body of evidence as well as continued review by independent regulatory agencies/committees including JECFA/Codex, FDA and EFSA^(9,21,22)^. These organisations have taken into account the decades of both positive and negative human and animal studies to draw their conclusions. Continual monitoring and modelling of LCS exposures is undertaken and this demonstrates that intakes of LCS, even among high consumers, are within ADI^(39–41)^.
(2) Currently, the major sources of LCS in the Western diet are beverages and table-top sweeteners^(39,40,42)^.
(3) LCS can be used to reduce the sugar and energy content of beverages and some foods whilst maintaining a similar sensory profile. The potential for energy reduction is more limited in foods and depends on the options for reformulation and what replaces the bulk of sugar^(43)^. LCS can be used synergistically in blends to achieve the desired sensory profile at lower levels of use.
(4) The collective evidence supports the conclusion that there is no relationship between adiposity and liking/preference for sweet taste in either adults or children^(44)^.
(5) Consumer perceptions vary with regard to LCS, with some individuals having concerns about their potential health effects^(14,20)^.

#### 2b Gaps: what we don’t know

(1) Which factors (including knowledge, attitudes and behaviours) influence consumer perception of risks and benefits of LCS consumption? Are these the same for health professionals?
(2) There is a need for in-depth data relating to current patterns of LCS consumption at multiple levels, and across countries and regions, to strengthen the evidence base.
(3) There is a need for more reliable measures of LCS exposure, such as biomarkers. Further development of these and better linkage of food composition and dietary databases are needed to help monitor changing use and consumption of LCS.

#### 2c Actions: what should be done?

(1) There is a need to research and develop evidence-based strategies to communicate all of the above to consumers, health professionals and policy makers. The extensive body of scientific evidence that backs regulatory approval and the ongoing safety assessment of LCS can then encourage better-informed public health decisions. The media or other organisations could be provided, for example, with simple explanations of the ADI.
(2) There is a need to develop communications to foster more informed public attitudes towards LCS, for example by emphasising the potential health gains associated with sugar (and energy) intake reduction and the role of LCS in achieving this. It is important to explain that the overall impact of LCS will depend on the amount of sugars replaced in the diet and the overall reduction in calorie (energy) intake that ensues. Use of LCS alone cannot be expected to act as a ‘silver bullet’ for weight loss.
(3) Research into biomarkers for individual LCS is needed to complement the exposure assessment based on consumption records. There is a need to improve linkage of databases and to model intakes in future scenarios.

### Theme 3: Role of low-calorie sweeteners in relation to nutrition policy

#### 3a Facts: what we know

(1) Reduction in the intake of ‘free sugars’ and ‘added sugars’ is being recommended around the world to reduce the risk and prevalence of obesity, which is a major public health concern^(10,45,46)^. LCS is one of the strategies to consider.
(2) LCS can be useful in dietary approaches to both prevent and manage diabetes and obesity. Benefit will depend on how foods and beverages containing LCS are substituted, as well as on the overall quality of the diet and the overall energy provision^(16)^.
(3) Despite repeated and consistent reassurances from food safety authorities, there is still some distrust of LCS among health professionals and policy makers^(47)^.
(4) Some policies acknowledge LCS consumption as a useful strategy to reduce sugars intake^(12)^. However, there are discrepancies with other national and international policies^(10,11)^ and regarding use in children.

#### 3b Gaps: what we don’t know

(1) Can LCS help individuals meet the population-level dietary recommendations for reduction of sugars intake (for example, to 5 % (average)^(48)^ or 10 % (for individuals)^(10,45)^)? If so, how can this be achieved?
(2) How does a dietary approach that includes LCS-sweetened foods and drinks affect dietary quality compared with low-sugar diets?
(3) What are the best strategies to communicate LCS safety and efficacy to interested parties such as health professionals and the general public?

#### 3c Actions: what should be done?

(1) There is a need to model the potential for LCS to reduce sugar content and sugar intakes whilst ensuring that other dietary recommendations can also be met in the overall diet.
(2) Trends in dietary intake of LCS need to be monitored, linked with food and beverage reformulation and ultimately with health outcomes.
(3) Policies relating to LCS from different countries should be reviewed to compare their remit, priorities, evidence base and interpretation.
(4) To help reconcile policy discrepancies, policy makers, scientists and regulatory affairs experts should agree on their understanding of the role of LCS in the diet.
(5) In the context of sugar reduction and obesity, it would be helpful to review the regulatory and public health policy hurdles that prevent wider use of LCS in food products for those sweeteners where dietary intake is very low compared with the ADI.

## Discussion

### Strengths and limitations of approach

The methodology followed a planned and transparent process. All seventeen experts were requested ahead of the workshop to generate a summary of their topic in the form of answers to the three questions. These were then collated under three themes by the workshop leaders and combined for the working document, which was circulated before the workshop. At the start of the group discussion, scoring was used as a consensus-forming technique to allow participants to indicate the strength of their agreement with each statement. Alternative forms of wording suggested by participants were considered in order to improve clarity of each statement. The resulting statements were circulated after the workshop, with supporting references, to allow for further reflection and improvement. A strength of the process was the expertise represented on the panel in many aspects of LCS (including toxicology, regulation, food science, medicine, microbiology, psychology, epidemiology, public health nutrition and dietetics). Finally, holding the workshop immediately after a scientific conference on the topic ensured that all experts were fully prepared and engaged to discuss the issues and formulate consensus.

The workshop was wide in scope but was not intended to be exhaustive; the themes were selected as being pertinent to current debate on LCS and within scope of the expertise of the panel. Consensus was based on expert opinion and key references including systematic reviews; the group did not review all the primary literature on these themes. Other possible limitations of the methodology were that all our participants were scientists or public health experts, unlike the broader stakeholder panel used by Bright *et al.*^(49)^. The workshop was instigated and funded by ISA; however, ISA had no control over the choice of themes, and no role in the discussion or this paper. Participants all acted completely independently to express their views in open debate and to contribute to the resulting paper.

### Comparison with other consensus papers relating to low-calorie sweeteners

#### Goals and methodology

To our knowledge, there have been three previous papers published in English that contain consensus statements about LCS^(14,15,49)^. In addition, there have been a number of position papers and evidence reviews whose methodology and scope differ from that of the present report and these are discussed later.

The goal of the consensus report by Gibson *et al.*^(14)^ was to summarise the role and potential benefits of LCS on appetite, energy intake, body weight, diabetes and dental health to give clarity to health professionals and educators on the use of LCS. The goal of the Ibero–American Consensus on LCS^(15)^ was ‘to develop a consensus on the use of low- and no-calorie sweeteners as substitutes for sugars and other energy sweeteners in line with current international public health recommendations, in the context of the prevention and treatment of obesity and related diseases in Latin American countries’. The report^(15)^ also provides a comprehensive overview of the position of international and national regulatory bodies on LCS safety and efficacy studies on individual LCS. Both these consensus reports^(14,15)^ were compiled by panels limited to international scientists and public health experts.

The report by Bright *et al*.^(49)^ focused on future research needs, and involved a wider stakeholder panel who participated in interactive webinars, surveys and interviews with the research team and generated a list of eighteen questions across five broad research areas, ranking them in order of priority. The stakeholder panel was recruited according to the ‘7 P’s’ of stakeholder engagement, i.e. patients, providers, researchers, policymakers, product makers, payers, and purchasers^(50)^. It therefore included policymakers, lay audience members, health providers, a research funder, individuals with food industry experience, and researchers of several different specialties.

#### Content and conclusions

The consensus statements agreed by our expert panel were produced independently but have been compared with previous consensus statements in Table 2. Further details can be found in online Supplementary Tables S1 and S2. Some topics were not covered in other reports: for example, the statements in this paper have included association between sweetness preference and obesity, effects of LCS on gut bacteria and sources of LCS, which were not covered by Gibson *et al.*^(14)^ or Serra-Majem *et al*.^(15)^; conversely, this panel did not consider the effects of LCS on dental health.

**Table 2.** Comparison of our consensus statements on low-calorie sweeteners (LCS) with those of others

Consensus statements	Gibson *et al.* (2014)^(14)^	Serra-Majem *et al.* (2018)^(15)^
Theme 1: Role of low-calorie sweeteners in weight management and glucose control: the scientific evidence		
1. When substituted for sugars to reduce energy density of foods and drinks, LCS can reduce net energy intake and assist weight management	+	+
2. Intervention studies have shown that LCS beverages have at least a similar effect on appetite and energy intake to water	+	
3. The collective evidence supports the conclusion that LCS have no adverse effect on blood glucose and insulin regulation (HbA1c, fasting and postprandial glucose and insulin levels) in individuals with, and without, diabetes	+	+
4. The potential value of LCS in dietary management of diabetes derives from their role as substitutes for sugars. and hence carbohydrates	+	+
5. Confounding by adiposity, and reverse causality can explain the positive association between LCS and type 2 diabetes and other cardiometabolic diseases, reported in some observational studies		+
6. Regarding effects involving the human gut microbiota, current evidence is limited and does not provide adequate evidence that LCS influence gut health at doses relevant to human use		
Theme 2: Consumption and safety of low-calorie sweeteners and consumer perception		
1. The safety of LCS is demonstrated by a substantial body of evidence as well as continued review by independent regulatory agencies/committees including JECFA/Codex, FDA and EFSA. These organisations have taken into account the decades of both positive and negative human and animal studies to draw their conclusions. Continual monitoring and modelling of LCS exposures are undertaken and this demonstrates that intakes of LCS, even among high consumers, are within ADI		+
2. Currently, the major sources of LCS in the Western diet are beverages and table-top sweeteners		
3. LCS can be used to reduce the sugar and energy content of beverages (and some foods) whilst maintaining a similar sensory profile. The potential for energy reduction is more limited in foods and depends on the options for reformulation and what replaces the bulk of sugar. LCS can be used synergistically in blends to achieve the desired sensory profile at lower levels of use	+	+
4. The collective evidence supports the conclusion that there is no relationship between adiposity and liking/preference for sweet taste in either adults or children		
5. Consumer perceptions vary with regard to LCS, with some individuals having concerns about their potential health effects	+	+
Theme 3: Role of low-calorie sweeteners in relation to nutrition policy		
1. Reduction in the intake of ‘free sugars’ and ‘added sugars’ is being recommended around the world to reduce the risk of obesity, which is a major public health concern. LCS should be one of the strategies to consider	+	+
2. LCS can be useful in dietary approaches to both prevent and manage diabetes and obesity. Benefit will depend on how foods and beverages containing LCS are substituted, as well as on the overall quality of the diet and the overall energy provision	+	+
3. Despite repeated and consistent reassurances from food safety authorities, there is still some distrust of LCS among health professionals and policy makers	+	+
4. Some policies acknowledge LCS consumption as a useful strategy to reduce sugars intake However, there are discrepancies with other national and international policies and regarding use in children		+

+, Broad correspondence with our consensus statements; blank, not (or not fully) addressed; JECFA, Joint Expert Committee on Food Additives; FDA, US Food and Drug Administration; EFSA, European Food Safety Authority; ADI, Acceptable Daily Intake.

Table 2 shows there was broad agreement between the sentiments expressed in our statements and these two reports.

The gaps identified by our panel have been compared with the research priorities from Bright *et al.*^(49)^ (online Supplementary Table S3). Most of the important future research questions identified by their stakeholder panel were also selected by our panel as areas in need of study. In the case of effects of LCS beverages on appetite and energy intake, our panel considered the evidence to be sufficiently strong for ‘no effect or at least similar effect’ compared with water to be classed as fact, and for a reduction in energy intake compared with sugar also to be classed as fact. Research gaps identified by our panel and not identified by Bright *et al*.^(49)^ included research on biomarkers of LCS consumption to aid intake assessments, research on communication with consumers and other stakeholders about LCS and more research on issues related to policy. Conversely Bright’s^(49)^ questions on the sensing of LCS by the brain and the impact of LCS on the fetus did not feature directly in our workshop discussion.

#### Comparison with other reviews and position statements

In 2011 EFSA was of the opinion that a cause-and-effect relationship had not been established between the use of intense sweeteners and maintenance of normal body weight or blood glucose, but several high-quality studies and reviews have since been published^(51–54)^ and others are currently underway: the SWITCH project^(55)^ and the SWEET project (available at https://sweetproject.eu). A number of reviews and position statements have addressed the evidence for and against health benefits of LCS. Our panel observed that differences between the positions and policies of different organisations with regard to LCS are a cause of confusion. Reasons for discrepancies may include different remits and approaches. The goal of systematic review and meta-analysis is frequently hampered by differing study designs that make comparison difficult and meta-analysis unreliable; hence the need for cautious wording, which may be interpreted as a negative statement. It is important to clearly establish that LCS are food additives and, as such, cannot provide health benefits, except in relation to the reduction of sugar within an adequate diet and lifestyle. Our panel concluded that, when used to replace dietary sugar, the use of LCS facilitates reduction in energy intake and weight loss. This was based on evidence from randomised controlled trials of 6 months to 2 years in length and recent systematic reviews that pay careful attention to appropriate comparators. The panel also stated the need for studies of longer-term effects. By contrast, a recent wide-ranging review on health effects of non-sugar sweeteners (which in practice were LCS as polyols were excluded) concluded that ‘there were no significant or clinically important effects on most outcomes’^(8)^. However, due to very strict inclusion and exclusion criteria, their analyses omitted some notable studies on body weight^(51–54)^ and combined studies with different comparators, potentially diluting the effect size^(56)^. Another recent review^(57)^ has been criticised on the same grounds^(37)^. Toews *et al.*^(8)^ also stated that ‘potential harms from the consumption of non-sugar sweeteners could not be excluded’, a statement which relates to lack of evidence, not evidence of harm. Our panel took a harm-reduction approach, where LCS are a desirable substitute for sugar and one route to helping achieve sugar and energy reduction whilst still maintaining dietary diversity and pleasure.

Other position statements, particularly those published before 2014, have offered cautious conclusions on potential benefits of LCS. For example, the 2012 joint scientific statement from the American Heart Association and the American Diabetes Association (AHA/ADA) concluded that ‘at present there are insufficient data to determine conclusively that non-nutritive sweeteners (NNS) benefit appetite, energy intake or body weight’^(20)^. However, the AHA/ADA document also stated that ‘when used judiciously, NNS could facilitate reductions in added sugars intake, thereby resulting in decreased total energy and weight loss/weight control and promoting beneficial effects on related metabolic parameters’.

The latest AHA advisory statement^(19)^ (which focused on LCS beverages and cardiometabolic outcomes) concluded that the use of LCS beverages may be an effective strategy to help control energy intake and promote weight loss. Nonetheless, due to the lack of long-term trials in children, the AHA thought it prudent to advise against prolonged consumption of LCS beverages by children, preferring water, other unsweetened beverages or milk as the primary drink. Policy statements from professional bodies of dietitians and nutritionists have generally been pragmatic, seeing LCS as a helpful tool in helping individuals reduce their sugar intake and manage their weight in the context of a healthy balanced diet that meets other dietary recommendations^(16–18)^.

#### Extension of our consensus statements to actions and policies

The main strategy of our consensus workshop was to stimulate forward thinking as well as to restate principles. The consensus statements on actions put the focus firmly on what is required to deliver. For example, the panel made recommendations for further long-term randomised controlled trials of LCS with different comparators and multiple endpoints, for prospective studies that control for adiposity and other confounders, and for better estimates of LCS exposure. Such recommendations may help research funding bodies select priorities. Clarity and consistency of policy would be improved by a comprehensive evaluation of all the evidence on effects of LCS. Others have also called for larger and longer clinical trials with careful selection of comparators^(7,37,44,58)^. The review by Toews *et al.*^(8)^ was also critical of the size, short duration, and methodological and reporting quality of studies. It also called for more data on benefits and risks of non-sugar sweeteners in doses and patterns more akin to real-life consumption^(8)^. Our expert panel considered the safety data to be robust but agreed that there is a continued need for ongoing exposure assessment to account for changing LCS use, and also consideration of any new evidence that might emerge. Novel recommendations made by the panel included better strategies and methods to improve communications about the safety and efficacy of LCS, modelling of the effect of LCS on sugar reduction and diet quality, relaxing regulation to increase the potential for reformulation using LCS, and review and reconciliation of policy differences on the use of LCS.

## Conclusion

The panel considered that the substantial body of evidence concerning LCS safety should be communicated in a consistent manner. More emphasis is required on the role of LCS in helping individuals reduce their sugar and energy intake, which is a public health priority.

Research priorities should include:

(a) a dossier of the totality of evidence on LCS and body weight control;
(b) studies to monitor and model LCS intakes and their impact on sugar reduction and diet quality;
(c) effective communication strategies to inform consumers, non-governmental organisations (NGOs), health professionals, research funding bodies and the food and beverage industry.

Efforts should be made to understand and, where possible, reconcile policy discrepancies between organisations and reduce regulatory hurdles that impede product development and reformulation designed to reduce sugars and/or energy.

It is hoped that these consensus statements and recommendations arising from the expert workshop will assist policy makers, and other stakeholders including NGOs, health professionals, research funding bodies and the food and beverage industry.

## Supplementary material

For supplementary material accompanying this paper visit https://doi.org/10.1017/S0954422419000283. click here to view supplementary material
