# Supplementary material for: Expert consensus on low-calorie sweeteners: facts, research gaps and suggested actions
Source: Nutr Res Rev. 2020 Jan 13;33(1):145–54. doi: 10.1017/S0954422419000283 (PMC7282854; doi:10.1017/S0954422419000283)
Supplement: Supplementary file 1 [file S0954422419000283sup001.docx]

# Supplementary information

## Comparison of Consensus statements from others with the Consensus statements in this paper

**Supplementary Table S1.** Conclusions from Gibson *et al.* (2014)

|  | Relevant to our consensus statement  Blank = not addressed |
| --- | --- |
| 1. LCS do not increase appetite and have no discernible effect on satiety | 1a2 |
| 1. LCS help to reduce energy when used in place of higher energy ingredients | 1a1 |
| 1. LCS can enhance weight loss under real-life conditions when used as part of a behavioural weight loss programme | 1a1 |
| 1. LCS may have a beneficial effect on post-prandial glucose and insulin in healthy individuals and in people with diabetes | 1a3  1a4 |
| 1. LCS have dental benefits when used in food, beverages, toothpaste and medications, provided other constituents are also non-cariogenic and non-erosive |  |

**Supplementary Table S2.** Conclusions from Serra-Majem *et al.* (2018)

|  | Relevant to our consensus statement  Blank = not addressed |
| --- | --- |
| (1) LNCS are some of the most extensively evaluated dietary constituents, and their safety has been reviewed and confirmed by regulatory bodies globally including the World Health Organisation, the US Food and Drug Administration and the European Food Safety Authority; | 2a1 |
| (2) Consumer education, which is based on the most robust scientific evidence and regulatory processes, on the use of products containing LNCS should be strengthened in a comprehensive and objective way; | 2b1  2a5 |
| (3) The use of LNCS in weight reduction programmes that involve replacing caloric sweeteners with LNCS in the context of structured diet plans may favour sustainable weight reduction.  Furthermore, their use in diabetes management programmes may contribute to a better glycaemic control in patients, albeit with modest results.  LNCS also provide dental health benefits when used in place of free sugars | 1a1  1a3  1a4 |
| (4) It is proposed that foods and beverages with LNCS could be included in dietary guidelines as alternative options to products sweetened with free sugars; | 2a3  3c1  3a4 |
| (5) Continued education of health professionals is required, since they are a key source of information on issues related to food and health for both the general population and patients. With this in mind, the publication of position statements and consensus documents in the academic literature are extremely desirable. | 3a3  3c4 |
|  |  |

**Supplementary Table S3.** Conclusions from Bright *et al.* (2017) on future research needs

|  | Relevant to our consensus statement  Blank = not addressed |
| --- | --- |
| Q1. Do LCSs aid weight loss and/or weight maintenance? | 1a1 1b1 |
| Q2. Does LCS consumption modify appetite (hunger, fullness, desire to eat /prospective consumption) and/or total energy intake and, if so, how? | 1a1, 1a2 |
| Q3. Does the use of LCSs affect insulin secretion, carbohydrate metabolism, or the gut microbiota and its function? If so, where is this happening (cognition, sweet receptors on tongue, receptors in gastrointestinal tract, etc.) and does it have any physiologic consequences on health? | 1b1 |
| Q4. Are there potential long-term health risks (obesity, diabetes, cancer, CVD, etc.) of LCS consumption in humans? Are certain population groups (diabetics, children, pregnant women, those with genetic disease) more susceptible to the potential health risk(s)? | 1b1, 1b2 |
| Q5. Is LCS sweetness perceived by the brain as energy in the same way as other sweeteners? Do those who are overweight or obese sense LCSs differently than normal-weight people? |  |
| Q6. Are there impacts of LCS consumption during pregnancy on the fetus? |  |
| Q7. Do LCSs differentially affect long-term food intake, eating frequency, and portion sizes in children, adolescents, and adults? Is there an impact on dietary quality and adherence to recommended dietary patterns? | 1b4?  3b1  3b2 |
| Q8. In individuals with diabetes and prediabetes, does chronic consumption of LCSs have an impact on glycemic control, alter glucose transport, or invoke a cephalic phase response? | 1b5 |
| Q9. Does LCS consumption affect consumption of other sweeteners or sugars or total carbohydrate intake? Is the effect different than that from consumption of nutritive sweeteners? | 1b4  2b3 |
| Q10. Do LCSs affect energy metabolism and fat storage? | 1b1 |
| Q11. Should study findings be evaluated for each LCS individually or collectively? To which health outcome(s) are the findings from individual LCSs generalizable to the class of ingredients? | 1b3 |
| Q12. Is LCS intake accurately estimated in current dietary assessment tools? | 2b3 |
| Q13. Are there interactions between the combination of fat substitutes and sweetener substitutes on appetite (hunger, fullness, desire to eat or prospective consumption) and/or total energy intake? | related to 1b2 |
| Q14. Is there any variation in how LCSs affect those of different ages, races, and ethnicities? | 1b2 |
| Q15. Do individuals with different dietary patterns (high protein vs. high carbohydrate, etc.) affect the metabolism of LCSs differently and, if so, how? | 1b2 |
| Q16. How do we design a system or methodology to address the differences in existing LCS compounds vs. compounds that will be emerging down the road? | 2a1? 2b2  2b3 |
| Q17. Do the effects of LCS consumption on body weight differ by sex? If so, what are the sex-specific mechanisms of the impact of LCS consumption on body weight? | 1b2 |
| Q18. Has there been a gradual increase in the overall sweetness in our diet? | related to 1b4 |
